# Childhood stunting is highly clustered in Northern Province of Rwanda: A spatial analysis of a population-based study

**DOI:** 10.1016/j.heliyon.2024.e24922

**Published:** 2024-01-19

**Authors:** Albert Ndagijimana, Gilbert Nduwayezu, Clarisse Kagoyire, Kristina Elfving, Aline Umubyeyi, Ali Mansourian, Torbjörn Lind

**Affiliations:** a Department of Clinical Sciences, Pediatrics, Umeå University, Umeå, Sweden; b University of Rwanda, College of Medicine and Health Sciences, School of Public Health, Kigali, Rwanda; c Department of Physical Geography and Ecosystem Science, Centre for Geographical Information Systems, Lund University, Lund, Sweden; d University of Rwanda, College of Sciences and Technology, Centre for Geographic Information Sciences, Kigali, Rwanda; e School of Public Health and Community Medicine, Gothenburg University and the Queen Silvia's Children Hospital, Gothenburg, Sweden

**Keywords:** Spatial, Child, Stunting, Undernutrition, Rwanda, Sub-saharan africa, LMICs

## Abstract

**Background:**

In Northern Province, Rwanda, stunting is common among children aged under 5 years. However, previous studies on spatial analysis of childhood stunting in Rwanda did not assess its randomness and clustering, and none were conducted in Northern Province. We conducted a spatial-pattern analysis of childhood undernutrition to identify stunting clusters and hotspots for targeted interventions in Northern Province.

**Methods:**

Using a household population-based questionnaire survey of the characteristics and causes of undernutrition in households with biological mothers of children aged 1–36 months, we collected anthropometric measurements of the children and their mothers and captured the coordinates of the households. Descriptive statistics were computed for the sociodemographic characteristics and anthropometric measurements. Spatial patterns of childhood stunting were determined using global and local Moran's I and Getis-Ord Gi* statistics, and the corresponding maps were produced.

**Results:**

The z-scores of the three anthropometric measurements were normally distributed, but the z-scores of height-for-age were generally lower than those of weight-for-age and weight-for-height, prompting us to focus on height-for-age for the spatial analysis. The estimated incidence of stunting among 601 children aged 1–36 months was 27.1 %. The sample points were interpolated to the administrative level of the sector. The global Moran's I was positive and significant (Moran's I = 0.403, p < 0.001, z-score = 7.813), indicating clustering of childhood stunting across different sectors of Northern Province. The local Moran's I and hotspot analysis based on the Getis-Ord Gi* statistic showed statistically significant hotspots, which were strongest within Musanze district, followed by Gakenke and Gicumbi districts.

**Conclusion:**

Childhood stunting in Northern Province showed statistically significant hotspots in Musanze, Gakenke, and Gicumbi districts. Factors associated with such clusters and hotspots should be assessed to identify possible geographically targeted interventions.

## 1 Introduction

Undernutrition, which has an estimated worldwide prevalence of 22 % among children aged under 5 years [1], remains a major contributor to child mortality and disease burden, mostly in low- and middle-income countries (LMICs); stunting, which is defined as a height-for-age z-score (HAZ) 2 standard deviations (SDs) below the reference value, is the most prevalent form (estimated prevalence, 22.3 %) [2]. Undernutrition (defined by the authors as stunting, wasting, foetal growth restriction, suboptimal feeding, and lack of zinc and vitamin A) is the cause of approximately 45 % of all child deaths [3]. Over the last two decades, the global prevalence of childhood undernutrition has decreased considerably, except in sub-Saharan Africa, which has seen an increase of 12.4 million cases, especially in the stunting form [4]. In sub-Saharan Africa, the pooled prevalence of stunting among children aged under 5 years is 33.2 %, especially in East Africa (39 %) and West Africa (31.8 %), lagging far behind the global nutrition targets of 2025 [5]. These findings highlight the need to invest in the first 1000 days of childhood to alleviate malnutrition, especially stunting [6], and remove the irreversible effects of malnutrition on child development and cognitive function [7,8], which hinder economic development [9].

The main conventional global determinants of childhood undernutrition are poverty, preterm birth or small-for-gestational age, repeated childhood infections exacerbated by poor sanitation and unsafe drinking water, food insecurity, limited access to health services, inadequate childcare and feeding practices, poor maternal health and nutrition, insufficient maternal education, short birth intervals, and large family sizes [10,11]. A longitudinal study conducted on a cohort of newborns in seven low-income countries from Asia, America, and South Africa identified lower enrolment weight-for-age, shorter maternal height, enteropathogenesis, lower socioeconomic status, and low protein intake as factors contributing to the odds of being in a lower length-for-age at 24 months [11]. However, only a small number of studies have investigated the very influential aspect of spatial epidemiology in childhood undernutrition [12,13]. In this regard, some studies conducted in East Africa, especially in Ethiopia, showed that childhood undernutrition was not randomly distributed but clustered, providing an argument to redirect nutritional interventions and address geographical inequalities with a focus on hot spots, that is, areas with a larger number of cases [[14], [15], [16], [17]].

In Rwanda, a landlocked country in eastern Africa, childhood malnutrition continues to pose a serious public health threat despite several interventions, including infant and young child feeding promotion programmes, annual national mother and child weeks, community-based nutrition programmes, behaviour change communication, and home-food fortification. The Rwanda Demographic and Health Survey (RDHS) of 2019–2020 showed that the prevalence of stunting among Rwandan children aged 6–59 months was 33%—a 4.4 % reduction from 2014 to 2015, while the prevalence of wasting (weight-for-height z-score [WHZ] 2 SDs below the reference value) was 1 %, that of underweight (weight-for-age z-score [WAZ] 2 SDs below the reference value) was 8 %, and that of overweight (heavy for their height) was 6 % [18].

A secondary analysis of the RDHS 2014–2015 data showed that male sex, age beyond 6 months, low maternal height, low level of maternal education, not taking deworming tablets during pregnancy, and low-income households were factors associated with stunting in children aged under 5 years [19]. Apart from individual- and household-related factors, very few studies have explored other aspects, such as spatial patterns of childhood undernutrition, to supplement the classical predictors of stunting. An analysis of the DHS data of 2014–2015 attempted this and found elevation to be a significant factor for low height-for-age in children aged under 5 years, but the spatial prediction map showed no variability of height-for-age at the cluster (village in DHS) level when the levels were aggregated at the district level [12].

Over the last two decades, Northern Province in Rwanda has shown the highest rate of stunting among children under the age of five, with the prevalence increasing from 39.2 % in 2014–2015 to 40.5 % in 2019–2020, in contrast to the decrease in prevalence at the national level during the same period [18]. This was again confirmed by Uwiringiyimana et al. who, in a Bayesian geostatistical modelling of stunting in Rwanda, found Northern Province on top with a higher risk of stunting even after accounting for other covariates in the spatial model [13]. Moreover, 55 % of stunted children under aged 5 years lived in highland areas (altitude ≥1641.5 m above sea level), a key topographical characteristic of Northern Province, and showed a 29 % higher risk of stunting than children living in the lowlands, independent of other risk factors [14]; this increased risk was associated with food unavailability in highlands and the high need for food intake by children living in these areas since they are subject to higher levels of physical activity than those from lowlands.

Nevertheless, the aforementioned studies on spatial analysis of childhood stunting in Rwanda did not assess its randomness and clustering, and none were conducted in Northern Province [20]. Therefore, in the present study, we conducted an in-depth exploration of the spatial patterns of childhood undernutrition from a population-based study on infants and children aged 1–36 months and their mothers in Northern Province with the aim of identifying vulnerable clusters, a key requirement for the implementation of targeted interventions in the fight against malnutrition in Rwanda.

## 2 Materials and methods

### 2.1 Study design

A population-based, cross-sectional study was conducted using a quantitative household questionnaire on childhood nutritional status, mother and child characteristics, and geographical coordinates in selected households with children aged 1–36 months in Northern Province with the assistance of the National Institute of Statistics of Rwanda, which provided a comprehensive and up-to-date list of enumeration areas (EAs) or clusters (villages in the Rwandan context) eligible for population research.

### 2.2 Study setting

**Fig. 1:**
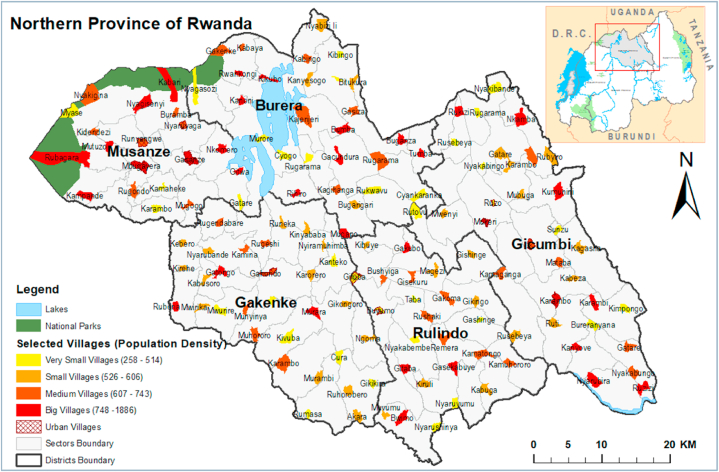
Selected villages (clusters) in the districts of Northern Province based on their population density, 2021.

Northern Province is one of the four provinces that, together with the city of Kigali, make up Rwanda. It contains five districts: Burera, Gakenke, Gicumbi, Musanze, and Rulindo. It is characterised by high altitudes and is a part of the mountains that form the Congo-Nile Crest (Fig. 1).

### 2.3 Study population

This study targeted children under three years of age, and a survey questionnaire was administered to the primary caregiver (mother) at the household level. We included households with a child aged below 3 years and excluded households with a mother younger than 18 years or a severely sick mother who could not respond to the questionnaire.

### 2.4 Sampling method and sample size

The sample size (n) was determined using the following formula for prevalence studies:

$$n=\frac{Z_{\propto /2}^{2}\times p\times \left(1-p\right)}{d^{2}}\times DEFF$$

where n is sample size, Z_α/2_ is the critical value of the normal distribution at α/2 (e.g., for a confidence level of 95 %, α is 0.05 and the critical value is 1.96), d is the degree of precision (0.05), p is the proportion of stunting in Northern Province (40.5 %) [18], and DEFF is the design effect. From the formula, n was determined to be 553.19.

After allowing for a 10 % nonresponse rate, we set the sample size to 615 households. A two-stage cluster sampling technique was used to select households. The first stage entailed randomly selecting 137 villages (enumeration areas) from the 5 districts of Northern Province. The second stage consisted of randomly selecting 615 households with at least one child aged 1–36 months for the survey; among them, 160 had to have livestock, that is, lactating cattle (approximately ¼ of all surveyed households). Different numbers of households were selected based on the village size. In the 69 very small and small villages, 3–4 households were visited, and in the 68 medium-sized and large villages, 5–9 households were visited (Fig. 1).

The sampling of villages was part of the spatial aspect of the study; that is, households were distributed across the whole district and within a similar distance between them. The list of households (sampling frame) was made available by community health workers in charge of maternal and child health at the village level. Systematic random sampling was performed to obtain the required number of eligible households within reasonable topographic distance to ensure sufficient representation across the village. Systematic random sampling was applied by dividing the number of eligible households by the required sample size in the cluster to obtain the sampling interval (I), randomly selecting the first household between the first and I^th^ household in the frame, then adding I to the first household to identify the next household, and so forth, until the sample size was reached. If a selected household was not found, replacement was performed with the nearest eligible household.

### 2.5 Data collection methods

The household questionnaire covered the sociodemographic characteristics of the household (e.g. possessions, Ubudehe category, education, and main occupation) and causes of undernutrition (e.g. source of drinking water, livestock possession, and milking cow possession). Anthropometric measurements (length/height, weight, head circumference, and mid-upper-arm circumference for children; height and weight for mothers) were obtained using locally produced length boards, digital weight scales (SECA AG, Hamburg, Germany), and tape measures. Household coordinates were captured using GPS. Data collection was performed by well-trained enumerators (university graduates from the health sciences) after a 1-week training in the city of Kigali and piloting in the Rulindo district.

### 2.6 Data analysis

#### 2.6.1 Child and mother nutritional analysis

This study aimed to assess the spatial distribution of undernutrition in children aged below 3 years in Northern Province, Rwanda, using a Geographic Information System (GIS). This study aimed to test the hypothesis that stunting is non-randomly distributed across different clusters in Northern Province.

Based on anthropometric measurements from children aged 1–36 months (weight in grams, height in centimetres, and age in months), z-scores were estimated and categorised with reference to the WHO child growth standards [21]. In the present paper, we defined stunting as HAZ below 2 SDs, wasting as WHZ below 2 SDs, and underweight as WAZ below 2 SDs, in accordance with the RDHS estimates of childhood nutritional status [18] and with reference to the WHO child growth standards [22] and the WHO anthropometric measurement analysis [23]. Accordingly, the plausibility of z-scores was also evaluated, whereby children with HAZ scores below 6 SDs or above 6 SDs, WAZ scores below 6 SDs or above 5 SDs, or WHZ scores below 5 SDs or above 5 SDs were flagged as invalid and removed from the analysis [23]. For children's mothers, the body mass index (BMI) calculated as weight (in kilograms) over height squared (in cm) and categorised as normal (18.5–24.9 kg/m^2^), thin (<18.5 kg/m^2^), or overweight (≥25 kg/m^2^) [24].

Several key sociodemographic characteristics were identified for both mothers and their children, including district, mother's age in years, and education level (less than primary for those who did not complete 6 years of education, primary for those who completed 6 years of education at the primary level, and secondary or higher for those who finished high school or university). The Ubudehe category was used as a proxy for household wealth, and it was categorised according to the Government of Rwanda guidelines [25]. Category 1 was defined by the absence of housing and the ability to rent, very often struggling to get food, and struggling to get basic household items such as soap, salt, and clothes. Category 2 was defined by the ability to rent a house, eat at least twice a day, and work for wages. Category 3 was defined by the presence of sufficient food and assets. Other characteristics included having livestock of any kind and milking cows.

Anthropometric measurements were analysed using descriptive statistics with central tendency measures (mean and standard deviation). In addition, frequencies and percentages were reported using Stata 17 software (StataCorp. 2021. Stata: Release 17).

#### 2.6.2 Spatial analysis

##### 2.6.2.1 Interpolating sample points to the sector administrative level

**Fig. 2:**
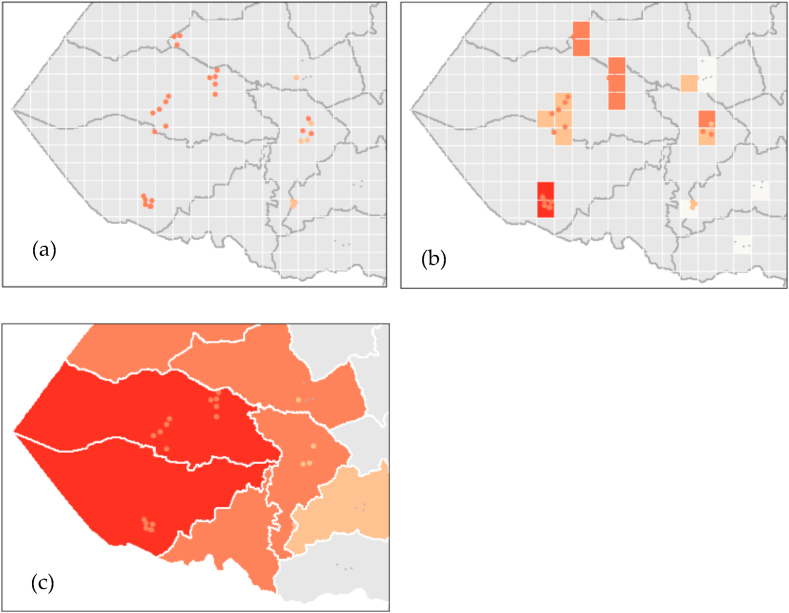
Sample interpolation. (a) Sampled households' point locations; (b) 1-km^2^ grids, (c) samples interpolated at the sector administrative level in Northern Province, 2021.

Point interpolation was used to construct a surface covering the study area [26,27]. As our sample formed small clusters at the village level, we generated a neighbour list from a point list before performing the autocorrelation analysis. Subsequently, we calculated the spatial weights for the neighbour lists. We first generated a 1-km grid, after which the samples inside that cell size were averaged into one value due to the geographic context of the study. We maintained the stunting pattern at the household level, which is logical because households within that area likely share similar living conditions. Otherwise, areas with clusters of observation points may dominate the interpolation of less-represented areas. Using the point interpolation method, we generated the areal stunting data at the sectoral administrative level [Fig. 2 (a - c)].

##### 2.6.2.2 Spatial autocorrelation analysis using global and local Moran's I values

We assessed the spatial patterns of undernutrition measures using global Moran's I statistic [28]. To determine the appropriate spatial weight that fit the data, we experimented with various cutoff threshold distances. This method was chosen because it is the most commonly recommended method for hotspot analysis (Getis-Ord Gi*) [29]. After multiple attempts, a threshold distance of 10 km was selected because it produced a higher global Moran's I value. Anselin local Moran's I was used to identify the concentrations of low and high undernutrition measures (local clusters) at a 95 % confidence level (95 % CI) [30]. Subsequently, hotspot analysis was performed using the Getis-Ord Gi* statistic to identify the locations of statistically significant hot- and cold-spots in the study area. The z-scores and p-values indicate locations where features with either high or low values are spatially clustered. The hotspot analysis based on the Getis-Ord Gi* statistic yielded a map showing statistically significant hotspots (areas with high stunting rates) and cold-spots (areas with low stunting rates). Clusters of areas with high stunting rates (hotspots) are indicated by high positive z-scores and low p-values (statistical probabilities), whereas low negative z-scores and low p-values indicate areas with low stunting rates. We computed global and local Moran's I statistics of stunting using ArcGIS Pro 3.0 (ESRI, Celartem, Inc.) and GeoDa (GeoDa Centre, University of Chicago).

## 3 Results

### 3.1 Sociodemographic characteristics

In total, 601 (97.7 %) of the 615 households with children aged 1–36 months were considered for analysis, while the remaining 14 had incomplete records. The households were almost evenly distributed across districts, with a slightly higher proportion of households (24 %) in Gicumbi (the largest district). The average age of the mothers of the participants was 32 years (SD, 7.2 years), with a majority of them aged 26–35 years (44.8 %), having no education (40.3 %), doing subsistence farming (69.7 %), and showing a normal nutritional status, i.e., BMI = 18.5–24.9 kg/m^2^ (71.3 %). A majority of the participants were from households within Ubudehe category 2, the poor category (48.6 %), and while a substantial proportion of these households had livestock of any type (84.2 %), only 26.0 % had milking cows (Table 1).

**Table 1:** Sociodemographic characteristics of children's mothers/caregivers in the Northern Province, 2021.

Characteristics	Frequency	Percentage
District		
Burera	129	21.5 %
Gakenke	130	21.6 %
Gicumbi	144	24.0 %
Musanze	96	16.0 %
Rulindo	102	17.0 %
Mother's age group (years)		
Mean (standard deviation)]	32 (±7.2)	
18–25	139	23.2 %
26–35	269	44.8 %
>36	192	32.0 %
Mother's education (n = 543)		
Less than primary	219	40.3 %
Primary level	193	35.5 %
Secondary or higher	131	24.1 %
Mother's main daily activity (n = 600)		
Housewife	132	22.0 %
Paid job	50	8.3 %
Agriculture	418	69.7 %
Maternal body mass index (n = 593)		
Normal (18.5–24.9 kg/m^2^)	423	71.3 %
Thin (<18.5 kg/m^2^)	23	3.9 %
Overweight (≥25 kg/m^2^)	147	24.8 %
Ubudehe category		
No category given	5	0.8 %
Ubudehe 1	63	10.5 %
Ubudehe 2	292	48.6 %
Ubudehe 3	241	40.1 %
Household has livestock		
No	95	15.8 %
Yes	506	84.2 %
Household has milking cow		
Yes	156	26.0 %
No	445	74.0 %
Total	601	100.0 %

### 3.2 Characteristics and nutritional status of children aged 1–36 months in Northern Province, Rwanda

The mean age of the children was 18 ± 10 months. In the assessment of nutritional status, 27.1 % of children were stunted (short for their age), 6.8 % were underweight (thin for their age), and 2.8 % were wasted (thin for their height/length) (Table 2).

**Table 2:** Children's nutritional status (z-scores) in Northern Province, 2021.

Characteristics	Frequency	Percentage
Age (in months)		
Mean (standard deviation)	18 (±10)	
1–11 months	199	33.1
12–23 months	213	35.4
24–36 months	189	31.5
Sex		
Male	290	48.3 %
Female	311	51.7 %
Stunting (HAZ < −2 standard deviations)	163	27.1 %
Underweight (WAZ < −2 standard deviations)	41	6.8 %
Wasting (WHZ < −2 standard deviations)	17	2.8 %
**Total**	**601**	**100.0 %**

HAZ, height-for-age z-score; WAZ, weight-for age z-score; WHZ, weight-for-height z-score.

**Fig. 3:**
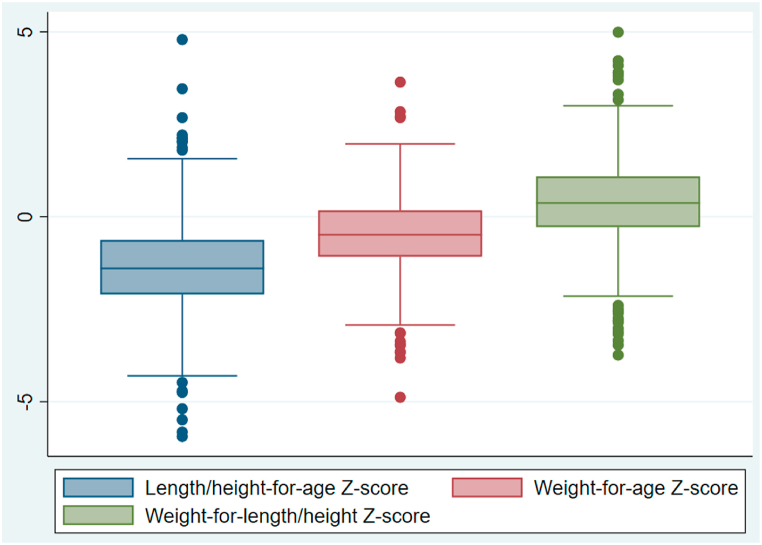
Box-and-whisker plot for anthropometric measurements (z-scores) for children aged 1–36 months in Northern Province, 2021.

Stunting increased with age (9.1 % at 0–6 months, 10.1 % at 7–11 months, 32.4 % at 12–23 months and 39.7 % at 24–36 months). Overall, the three anthropometric measurements (HAZ, WAZ, and WHZ) were normally distributed with almost the same variance (homogeneity), and the same trend was reported across all five districts of the Northern Province. However, the HAZ score was generally lower than those of the other two measurements, justifying the interest in the HAZ for spatial analysis. The mean scores were −1.37 (SD, 1.27) for HAZ, −0.48 (SD, 1.03) for WAZ, and 0.36 (SD, 1.16) for WHZ (Fig. 3). The analysis of variance (ANOVA) showed a low mean HAZ score variation between the five districts (Fisher's test = 1.64), which was not statistically significant (p = 0.1618).

### 3.3 Selection of adequate spatial weights

**Fig. 4:**
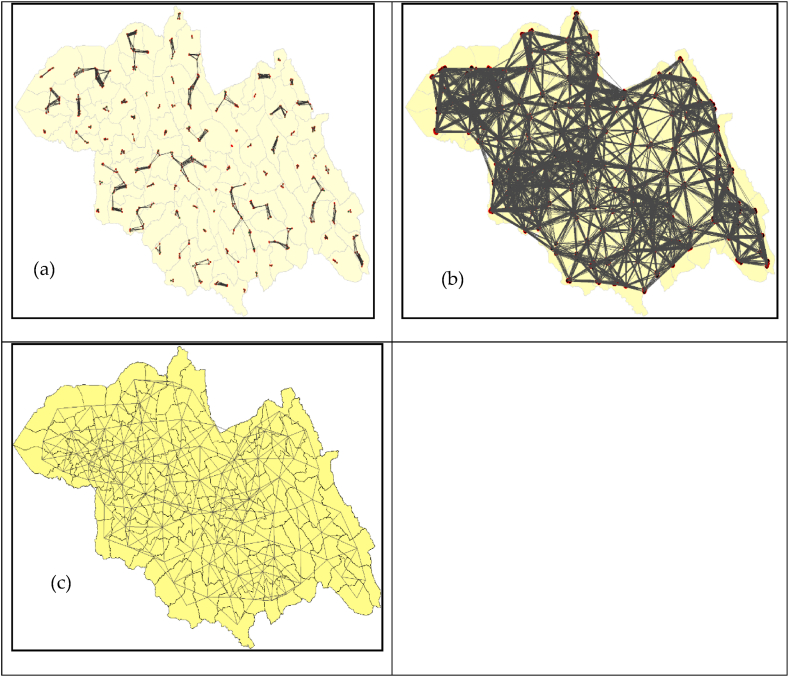
Spatial weights for neighbour lists with different threshold distances. (a) Using 3000 m as the cut-off distance, (b) using 10,000 m as the cut-off distance, and (c) neighbour list from the polygon list, Northern Province, 2021.

The spatial weights for neighbour lists at different threshold distances showed that the 3-km cutoff distance did not fit the data. Small sample clusters were grouped rather than connected to samples from adjacent clusters. However, the samples in one cluster had the ability to interact with the neighbouring samples at a threshold distance of 10 km. In the fixed-distance method, a feature is either a neighbour (1) or not (0). In the weighted method, neighbouring features have varying amounts of influence, and weights are computed to reflect this variation. Considering the size of the sectors in the study area, a threshold of 10 km was considered realistic [Fig. 4 (a - c)].

### 3.4 Global, local Moran's I and hot spot analysis for stunting

**Fig. 5:**
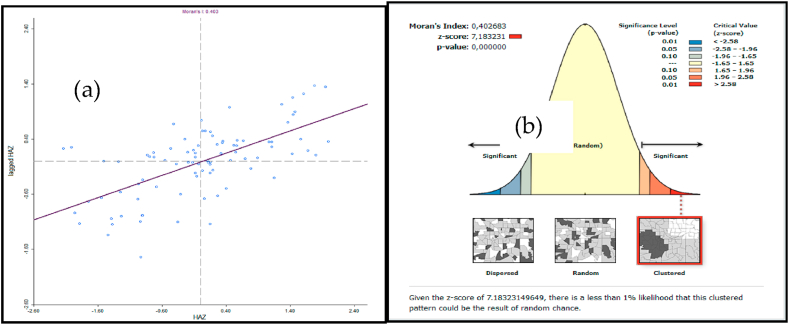
Stunting global Moran (a) scatter plot and (b) values, Northern Province, 2021.

The global Moran's I was positive and significant, suggesting a clustered pattern of stunting across the study area (Moran's I = 0.403, p < 0.001, z-score = 7.813), indicating a low likelihood that the spatial pattern was the outcome of a random process and a less than 1 % likelihood that the clustered patterns identified could be the result of random chance [Fig. 5 (a, b)].

**Fig. 6:**
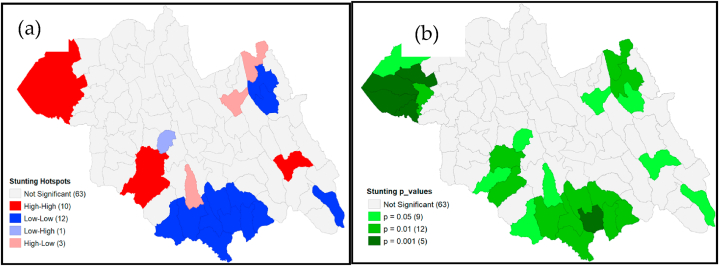
Stunting Local Moran's I significance (a) cluster map and (b) using p-values, Northern Province, 2021.

The local Moran's *I* classified the sectors as high-high clusters (n = 10), indicating high-risk clustering (hotspots); low-low clusters (n = 12), indicating low-risk clustering (cold-spots); not significant (n = 63); high-low clusters (n = 3), indicating low-risk sectors surrounding a high-risk sector; and low-high clusters (n = 1), indicating high-risk sectors surrounding a low-risk sector, using the p values = 0.05 (n = 9), p value = 0.01 (n = 12), not significant (n = 63), and p value = 0.001 (n = 5) [Fig. 6 (a, b)].

**Fig. 7:**
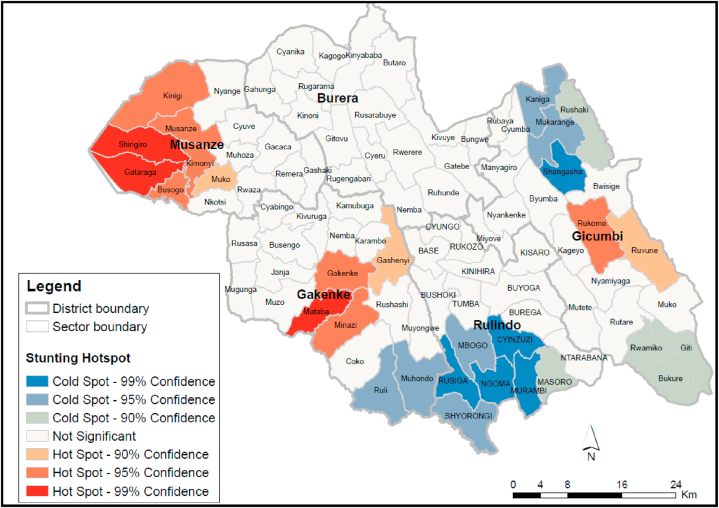
Stunting hotspot analysis (Getis-Ord Gi*) using confidence levels, Northern Province, 2021.

Based on the results of the hotspot analysis using the Getis-Ord Gi* statistic (Fig. 6), each sector was assigned a confidence level bin (Gi-Bin). This analysis showed that stunting was strongly clustered in Musanze (seven sectors), Gakenke (four sectors), and Gicumbi (two sectors) districts. Very low rates of stunting (cold-spots) were identified in the Rulindo (nine sectors) and Gicumbi (five sectors) districts (Fig. 7).

## 4 Discussion

This study aimed to assess the spatial distribution of undernutrition in children aged below 3 years in Northern Province, Rwanda. Spatial analysis showed that stunting was not randomly distributed; rather, it was clustered across different sectors of Northern Province. Furthermore, we found significantly high rates of stunting (hot spots) in Musanze, Gakenke, and Gicumbi districts, whereas significantly low rates of stunting (cold-spots) were found in Rulindo and Gicumbi districts. Our results are different from those of a study that assessed the spatial pattern of stunting in Rwanda using DHS data and found a lack of variability in height-for-age with aggregation at the district level [12]; this may be attributable to the scarcity of geographic points in the DHS (500 points in all 30 districts of the country), while our study gathered point records from 601 households in 5 districts. The only other study on stunting in Northern Province, specifically in Musanze, one of its five districts, did not assess its randomness or clustering [20]. Spatial clustering of childhood stunting has also been found in other low-middle-income countries, such as India [31], Ethiopia [14,15,17,32,33], Nepal [34], Pakistan [35], and Ghana [36].

The presence of significant hot- and cold-spots of childhood stunting remains a common trend across different countries that have assessed its spatial patterns, such as India [31], Ethiopia [14,15,17,33], Pakistan [35] and Ghana [36]. The most likely explanation for these patterns is shared risk factors across clusters, not the geographical divisions *per se*. In the present study, many factors could explain the clustering and hotspots. Food security and socioeconomic resources in this region are generally low. Although Musanze district is one of the top granaries of the country, especially in terms of potatoes and other vegetables, and cereals, a large proportion of the crops are sold to markets, and only 40 % are consumed at home, indicating the possibility of low food quantity in households in Musanze and Burera districts [37]. The Gicumbi and Gakenke districts are also fertile and have high rainfall throughout the year, which is favourable for cropping and food security. A study conducted among children aged 5–30 months in Northern Province identified exclusive breastfeeding as protective against stunting, and the caregiver's BMI was positively associated with the child's HAZ scores [20]. Poor infant feeding practices among caregivers, especially complementary feeding and diet diversity, lower exclusive breastfeeding during the first 6 months, low levels of knowledge among caregivers, and low socioeconomic status to buy animal-derived foods, have been highlighted as some of the reasons behind childhood stunting in Northern Province [20]. In Musanze district, the percentages of minimum dietary diversity, minimum acceptable diet, and consumption of iron-rich foods were 57 %, 53 %, and 29 %, respectively [38]. Environmental factors such as elevation have also been found to affect the height-of-age of children in Rwanda [39], whereas the effects of other climatological factors are virtually unknown.

In India, district-level variabilities in terms of extreme temperatures and the level of per capita crop production explain the existence of strongly significant hotspots in some districts [31]. In Ethiopia, rural regions are more likely to have strong stunting hotspots for different reasons, including high poverty, whereby children do not get sufficient calorie intake [14], population density, climate, and childhood diseases [17]. In Pakistan, poverty, low exposure to mass media among women, large family sizes, and extreme climatic factors (flooding or drought) have been found to explain stunting hotspots in some areas [35]. In Ghana, high fertility, low access to healthcare services, poverty, and illiteracy stood out as explanations for higher rates of stunting [36].

In the present study, the prevalence rates of stunting, underweight, and wasting were 27.1 %, 6.8 %, and 2.8 %, respectively. Overall, the prevalence of stunting was lower than that reported in the recent RDHS of 2019–2020, which estimated a prevalence rate of 39.9 % among children aged 1–36 months. This difference may have been due to differences in sampling techniques, with the spatial focus of our study allowing it to reach out to households from all the corners of the study area. For instance, this study and the RDHS showed similar prevalence rates during the first six months of life (18.2 %) in Northern Province, but this study showed a lower prevalence in children aged 7–11 months (10.1 %). A few studies support the idea that some children show stunting immediately after 6 months due to non-optimal feeding practices [[40], [41], [42], [43]]. A cross-sectional study in Musanze district reported that 62 % of children had low dietary diversity in complementary feeding [20], emphasising that poor complementary feeding practices are the strongest predictors of stunting [44,45], especially in Africa and Asia [46].

The mean HAZ score was −1.4 ± 1.3, a relatively lower value than that reported in the RHDS 2014–15 [12]. This study found a gradual increase in stunting with age, which is similar to the findings in other studies conducted in Rwanda [12] and elsewhere [47].

### 4.1 Strengths and limitations

This study provides a detailed depiction of the spatial patterns of childhood stunting at a low administrative level, the first of its kind in Rwanda. The study captured geographical coordinates at the household level, allowing a very precise geospatial analysis in contrast to previous studies, and was more in-depth than the usual summaries across larger geographical areas. Thus, this study is unique in its ability to ascertain the spatial clustering of stunting at the sectoral level in Northern Province in Rwanda. Furthermore, a representative sample of the population was recruited using sound methodology, providing high external validity to the findings. However, the spatial autocorrelation at the initial cluster level, that is, below the sector level, was weak, probably because there were too few households during sampling. In addition, although mothers younger than 18 years of age were excluded from the study as 18 years was the threshold age for consent in Rwanda, these mothers also lived in difficult conditions that could have resulted in undernourished babies, such as single-head households and large family sizes [45]. However, the RDHS 2019–2020 estimated that less than 5 % of mothers in Rwanda were teenagers (aged 15–19 years) [18]; therefore, their exclusion did not considerably affect the validity of the study findings. Finally, this study was purely descriptive and did not show factors that were statistically associated with the clustering of stunting in Northern Province, Rwanda. Further studies are necessary to investigate the factors associated with these hotspots for spatially or geographically focused, evidence-based interventions.

## 5 Conclusions

Childhood stunting, which showed an estimated incidence of 27.1 % in Northern Province, Rwanda, represents a major threat to the future of the affected children. Stunting was found to be clustered across different sectors of Northern Province, with Musanze, Gakenke, and Gicumbi districts being statistically significant hotspots. Our findings highlight the need for further assessment of the factors associated with the clustering of childhood undernutrition as well as their distribution across different sectors for specifically guided interventions. The findings also call for geographically targeted interventions with a focus on areas (sectors) with childhood stunting hotspots, in contrast to the routine approach of undernutrition alleviation in Rwanda and similar settings.

## Ethics statement

This multidisciplinary study involved eight doctoral students and two postdoctoral fellows. Before data collection, ethical approval was obtained from the University of Rwanda College of Medicine and Health Sciences Institutional Review Board (IRB), with Reference N^o^ 295/CMHS/IRB/2022, unique to multidisciplinary teams. All the participants provided informed consent to participate in the study. All information obtained from the respondents was kept confidential. Participation in this study was voluntary. The study participants did not have to respond to any questions they felt uncomfortable with, and they could withdraw from the study at any time without any negative consequences.

## Funding

This study was funded by the Swedish International Development Cooperation Agency (SIDA), grant number (SIDA Contribution no 11277), especially for data collection.

## Availability of data and materials

The datasets generated and/or analysed during the current study are not publicly available because of the privacy of the participants (their personal identity and other very sensitive information). The large dataset hosts records from eight multidisciplinary projects, including gender-based violence and mental health) and is available from the corresponding author upon reasonable request.

## CRediT authorship contribution statement

**Albert Ndagijimana:** Writing – review & editing, Writing – original draft, Visualization, Validation, Software, Methodology, Investigation, Formal analysis, Conceptualization. **Gilbert Nduwayezu:** Visualization, Validation, Software, Methodology, Formal analysis. **Clarisse Kagoyire:** Validation, Methodology. **Kristina Elfving:** Writing – review & editing, Validation, Supervision. **Aline Umubyeyi:** Writing – review & editing, Validation, Supervision. **Ali Mansourian:** Writing – review & editing, Validation, Supervision. **Torbjörn Lind:** Writing – review & editing, Validation, Supervision, Methodology, Conceptualization.

## Declaration of competing interest

The authors declare that they have no known competing financial interests or personal relationships that could have appeared to influence the work reported in this paper.

## List of abbreviations

ANOVA: Analysis of variance
BMI: Body Mass Index
CI: Confidence Interval
GIS: Geographic Information System
GPS: Global Positioning System
HAZ: Height-for-Age z-score
IRB: Institutional Review Board
LMICs: Low- and middle-income countries
RDHS: Rwanda Demographic and Health Survey
SD: standard deviation
WAZ: Weight-for-Age z-score
WHO: World Health Organization
WHZ: Weight-for-Height/length z-score
